# Mumps and Splenic Abscess: Is There a Link?

**DOI:** 10.7759/cureus.33195

**Published:** 2022-12-31

**Authors:** Jyotsnaa Muralitharan, Vijayakumar Nagarajan, Umarani Ravichandran

**Affiliations:** 1 Internal Medicine, Rajah Muthiah Medical College & Hospital, Chidambaram, IND

**Keywords:** multi-organ disease, tropical infectious diseases, vaccine-preventable diseases, hydroureteronephrosis, parotitis, pneumonitis, myocarditis, splenic abscess, mumps

## Abstract

Mumps is an acute viral illness occurring in children and young adults transmitted via droplets. It is a vaccine-preventable illness caused by the mumps virus, an RNA (ribonucleic acid) virus belonging to theParamyxoviridaefamily. It typically presents with fever, parotitis, epididymo-orchitis, oophoritis, meningitis, encephalitis, pancreatitis and arthritis. Although viremia with multiorgan involvement is known to be commonly seen in mumps, there have been no reported cases of splenic abscess in a case of mumps.

Here we present the case of a 16-year-old girl with unknown vaccination history who presented with fever, rash, bilateral parotid swelling, myocarditis, pneumonitis with pleural effusion and shock. Enzyme-linked immunosorbent assay (ELISA) for mumps Immunoglobulin M (IgM) antibody was positive (ratio: 7.26, reference: 1.10). She was managed conservatively with parenteral antibiotics, oxygen, inotropic support and bronchodilators. As she complained of abdominal pain in the left hypochondrium on the 13th day since onset of symptoms, ultrasound scan of abdomen was done which showed a hypoechoic lesion with internal echoes in the inferior pole of spleen (2.9 cm x 2.2 cm) suggestive of splenic abscess. Computed tomography (CT) of abdomen confirmed similar findings. The splenic abscess completely regressed with parenteral antibiotics. Therefore, one must suspect splenic abscess in a case of mumps when the presentation includes abdominal pain and tenderness so that appropriate treatment may be provided for the best outcome for the patient.

## Introduction

Mumps, a vaccine-preventable disease transmitted via droplets, is caused by mumps virus, an RNA virus belonging to the family Paramyxoviridae [1]. It is known to present as an acute infectious illness in children and young adults with fever, parotitis, epididymo-orchitis, oophoritis, meningitis, encephalitis, pancreatitis and arthritis [2-4]. It has an incubation period of 15 to 24 days [5], with the viral transmission being greatest in the week preceding onset of parotitis. 

Approximately 75% cases of mumps are uncomplicated [6], presenting with fever, headache, vomiting, generalized aches and parotid gland enlargement. Among the cases with complications, aseptic meningitis is most common in children. Other complications include oophoritis (which may lead to sterility if bilateral) and epididymo-orchitis in adolescent boys, meningitis, encephalitis (0.5%), pancreatitis (3%) in older children and adults and deafness in a rare few cases [6-8].

Splenic abscess as a complication of mumps has not been reported in the literature, although pancreatitis complicated by pancreatic abscess has been described [9]. Here we report a case of mumps presenting with splenic abscess.

## Case presentation

**Figure 1:**
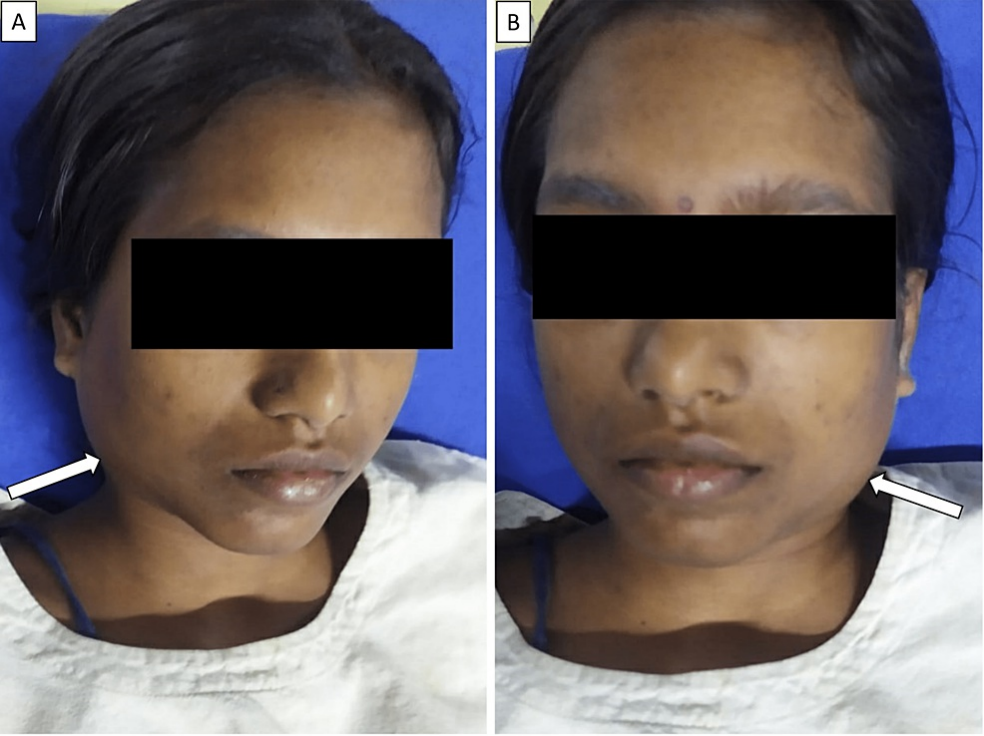
Bilateral parotid swelling (arrows)

A 16-year-old girl with unknown vaccination history, a previously healthy and immunocompetent individual, presented to the Emergency Room of our tertiary hospital in South India with a history of high-grade fever with chills and rigors for 10 days, productive cough for 10 days, bilateral parotid swelling for one week (Figure 1A, 1B), facial puffiness and pedal edema for five days. On examination, she was febrile (102 ℉), tachycardic and tachypnoeic. She had a blood pressure of 80/30mmHg and oxygen saturation of 90% in room air. She had a generalized erythematous rash, bilateral pedal edema, conjunctival congestion and bilateral parotid swelling. No eschar was seen. Right submandibular lymphadenitis was seen. Chest auscultation revealed wheeze and coarse crackles in the base of both lungs. Her abdomen was soft with no apparent organomegaly. There was no joint swelling or presence of subcutaneous nodules.

**Figure 2:**
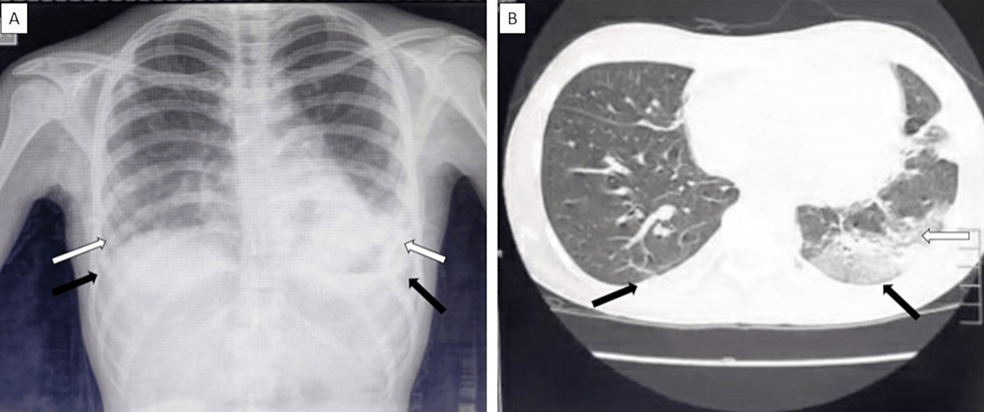
Chest X ray and CT thorax images showing bilateral lower lobe consolidation (white arrows) and pleural effusion (black arrows)

Initial investigations revealed a thrombocytopenia of 78,000/μL, total white blood cell count of 5900/μL and elevated erythrocyte sedimentation rate (ESR). Her liver function test, renal parameters and serum electrolytes were within normal range. Electrocardiogram showed sinus tachycardia. Chest X-ray showed bilateral lower zone opacification and blunting of costophrenic angles (Figure 2A). Investigations for enteric fever, leptospirosis, dengue fever, malaria, scrub typhus, human immunodeficiency virus (HIV) and melioidosis were negative. Blood culture did not grow any organism. Sputum smear for acid-fast bacilli, sputum culture and polymerase chain reaction (PCR) yielded negative results. Enzyme-linked immunosorbent assay (ELISA) for mumps Immunoglobulin M (IgM) antibody was found to be positive at a ratio of 7.26 (reference value: ratio of more than 1.10 is a positive test). Computed tomography (CT) of thorax done the next day showed bilateral lower lobe consolidation with bilateral mild pleural effusion (Figure 2B).

**Figure 3:**
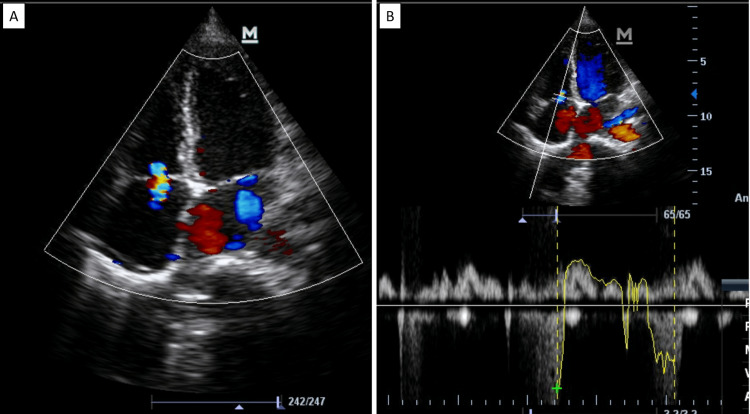
Echocardiogram with apical four chamber view showing dilated left atrium (LA) and left ventricle (LV), global hypokinesia of LV with moderate LV systolic dysfunction

Echocardiogram showed dilated left atrium and left ventricle, with global hypokinesia of left ventricle with moderate left ventricle systolic dysfunction (ejection fraction: 39%) suggestive of myocarditis (Figure 3A, 3B). Moderate mitral regurgitation was also present. No vegetations were visualized. Her creatine kinase-MB (CK-MB) was elevated at 54 IU/L.

She was admitted to the Intensive Care Unit and managed with injection piperacillin-tazobactam (4 gm i.v. every six hours), tab. doxycycline (100 mg twice daily), intravenous fluids, antipyretics, oxygen via face mask, inotropic support (norepinephrine) and nebulisation with bronchodilators.

**Figure 4:**
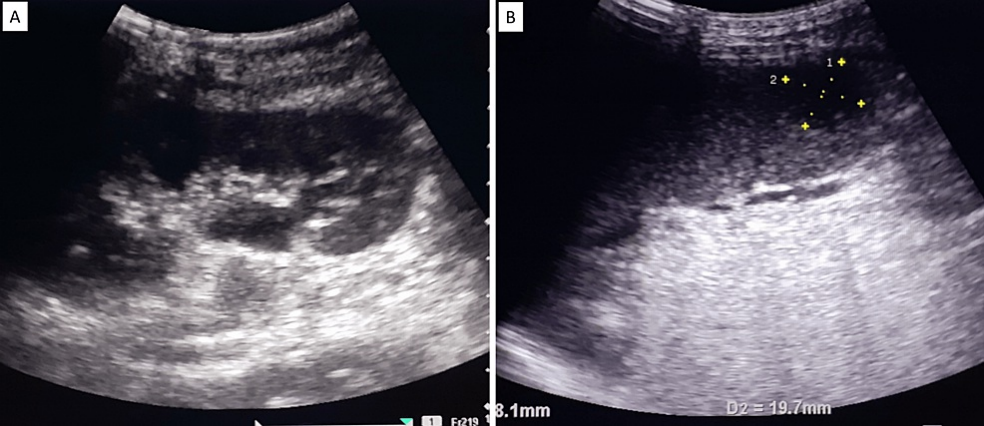
Ultrasound abdomen showing dilated pelvicalyceal system suggestive of hydroureteronephrosis (A) and a hypoechoic lesion with internal echoes in the inferior pole of the spleen suggestive of splenic abscess (B)

As the patient complained of pain in the left hypochondrium on the third day of admission, ultrasound abdomen was done which showed bilateral mild hydroureteronephrosis (Figure 4A) and a hypoechoic lesion with internal echoes in the inferior pole of spleen suggestive of splenic abscess (2.9 x 2.2 cm) (Figure 4B).

**Figure 5:**
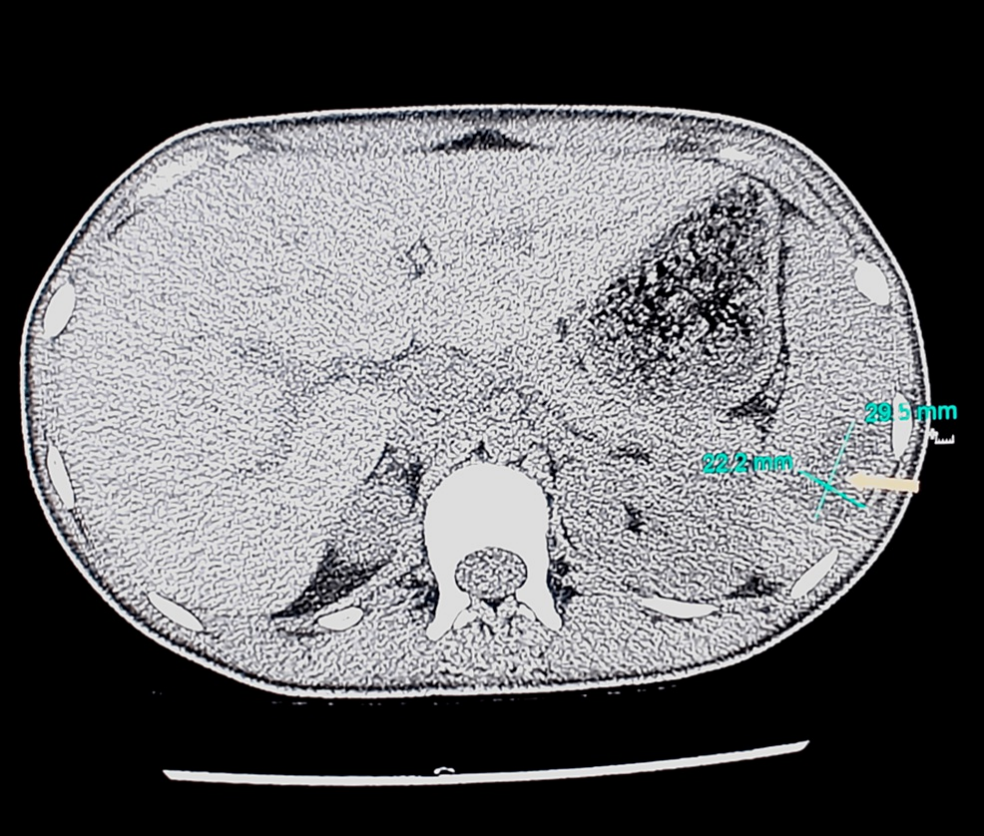
CT abdomen showing abscess in inferior pole of spleen of size 2.9 cm x 2.2 cm (arrow)

CT scan of abdomen confirmed the findings seen on ultrasound (Figure 5). A surgical consult was obtained for the same and antibiotics were continued as advised.

With the above treatment, the patient became afebrile on the fourth day since admission. Her oxygen saturation improved and shock recovered. Her platelet counts also improved. Following this, she was transferred out to the Intermediate Care Unit (IMCU) for continued management. However, abdomen pain and tenderness persisted. With continuation of antibiotics, the abdominal pain progressively reduced and the splenic abscess had reduced in size on the follow-up ultrasound scan. She was pain-free by day 14 and the abscess had completely regressed with 18 days of parenteral antibiotics. The patient was discharged on oral antibiotics and advised follow-up and vaccination with MMR (measles, mumps, rubella) vaccine. Echocardiogram done on follow-up after six weeks showed recovered left ventricle function with ejection fraction of 50%.

## Discussion

The classical presentation of mumps includes parotitis which is seen in 60-70% of infections and 95% of symptomatic patients [10]. The submandibular and sublingual glands are less commonly affected.

Besides manifestations like pancreatitis [9], meningitis, encephalitis, permanent unilateral sensorineural deafness, spontaneous abortion, and sterility in men following orchitis, rarer manifestations also occur. These rarer manifestations include myocarditis, arthritis, nephritis, hepatitis, thrombocytopenia, keratouveitis and hemophagocytic syndrome [11]. However, there have been no reported cases of splenic abscess in mumps.

The present case had fever, rash, parotitis, myocarditis, pneumonitis with pleural effusion, shock, hydroureteronephrosis and, in addition, a splenic abscess.

As literature on splenic involvement in mumps is lacking, the exact mechanism of splenic abscess is unclear. However, it is known that devitalized splenic parenchyma or infarcted splenic tissue is susceptible to abscess formation. Moreover, immunocompromised patients such as those with malignancy, HIV infection, postchemotherapy or posttransplant patients are the groups most commonly presenting with splenic abscess [12]. In these patients, any infection results in the circulating microorganisms seeding the splenic tissue causing an abscess formation. The organisms most commonly isolated from abscess fluid are gram-positive cocci Staphylococcus epidermidis and Staphylococcus aureus [12]. Next in frequency are gram-negative organisms like *Klebsiella *and *Enterobacter*. In patients with HIV, unusual organisms like Candida, Pneumocystis carinii, and Mycobacterium species have been identified [12]. Recently, splenic abscesses have been reported in cases of melioidosis [13]. Posttraumatic splenic abscesses have also been known to occur. Splenic abscess presents as fever, abdominal pain, splenic enlargement and tenderness. 

In the case of mumps infection, plasma viremia and affinity of the mumps virus to infect mononuclear cells has been known to cause the systemic spread of the virus with involvement of various organs including parotid glands, testes, pancreas and CNS [11,14-17]. Interstitial edema causing a pressure effect resulting in testicular atrophy [18] and proliferating necrotizing changes resulting in abortion in mumps-infected mothers have been described [19]. Similar spread to the mononuclear cells of the spleen and involvement of the organ by necrotizing changes and pressure effect of edema could have resulted in devitalization of splenic tissue, potentially providing a bed for inoculation by microorganisms. Our patient, being in an immunocompromised state, seems to have acquired a secondary bacterial infection, which has seeded the spleen resulting in splenic abscess. This is evident from the fact that the splenic abscess completely regressed in size with antibiotic treatment covering the common organisms implicated.

Diagnosis of mumps is based on isolation of virus or detection of viral nucleic acid or serological diagnosis (high titres of mumps IgM). Treatment for mumps is supportive including management of complications. Diagnosis of splenic abscess is by computed tomography (CT) where it is seen as a low-attenuation lesion. Presence of gas within the lesion is diagnostic for an abscess, although it is not visible in the majority of cases. CT helps identify the size and location of abscess. Treatment options for splenic abscess include medical therapy, percutaneous drainage and splenectomy. Medical therapy with antibiotics is preferred so long as the organism is susceptible to it. Percutaneous drainage may be employed when medical therapy is not effective, but it comes with the risk of recurrent infection. Splenectomy is preferred when there are multiple abscesses or if the suppuration has spread into the splenic vein, but it is best avoided in a young patient. 

Prevention by vaccination has a key role in mumps. Mumps vaccine is commonly administered along with measles and rubella vaccine as MMR vaccine. A single dose of vaccine decreases incidence of mumps by 88-98% and the booster dose decreases incidence by 97-99% [20]. If mumps does occur in a vaccinated individual, complications are less common. Post-exposure vaccination is not effective in mumps. However, vaccination is needed in these cases to prevent further infections.

## Conclusions

Mumps, while it is known to involve multiple organs, can also involve the spleen, resulting in a splenic abscess as a rare manifestation. The pathogenesis of splenic abscess involves viremic spread, affinity to mononuclear cells, tissue atrophy via the pressure effect of interstitial edema, proliferative necrotizing changes and secondary bacterial infection. Splenic abscess presents with fever, abdominal pain, splenic enlargement and tenderness.

Diagnosis of splenic abscess is by computed tomography which can also aid in identifying the size and location of abscess. Treatment is by medical therapy with antibiotics. Prevention has a significant role in mumps as vaccination can prevent the illness or reduce its severity.
